# Assessing work-related criticalities: psychometric properties of a questionnaire developed in an Italian occupational stress center

**DOI:** 10.3389/fpubh.2026.1791989

**Published:** 2026-05-08

**Authors:** Stefano Livellara, Anna Comotti, Elisa Carena, Matteo Bonzini, Giovanna Castellini

**Affiliations:** 1 Occupational Medicine Unit, Fondazione IRCCS Ca’ Granda Ospedale Maggiore Policlinico, Milan, Italy; 2 Department of Public Health and Pediatric Sciences, University of Turin, Turin, Italy; 3 Department of Clinical Sciences and Community Health, University of Milan, Milan, Italy

**Keywords:** occupational health, questionnaire validation, workplace adversity, workplace bullying, work-related stress

## Abstract

**Objective:**

Work-related stress and workplace adversity are increasingly recognized as major occupational health risks, particularly within complex organizational contexts. This study aimed to evaluate the psychometric properties of a newly developed instrument, the Work-Related Criticalities Questionnaire (WRCQ), designed to assess a broad range of work-related criticalities beyond traditional workplace bullying, within the Italian occupational setting.

**Methods:**

A retrospective analysis was conducted on 317 workers evaluated between 2021 and 2025 at an Italian Center for Occupational Stress. Participants completed the 39-item WRCQ together with measures of workplace bullying (NAQ-R), psychological distress (SCL-90-R), and anger expression (STAXI). Internal consistency, convergent and construct validity were examined. Confirmatory factor analyses were performed to test the multidimensional five-factors structure of the questionnaire, comprising interpersonal conflict, interpersonal hostility, organizational dysfunctions, organizational constraints, and professional devaluation.

**Results:**

The WRCQ demonstrated excellent internal consistency (Cronbach’s alpha = 0.91), with acceptable to good reliability across subscales. Convergent validity was observed with the NAQ-R (*r* = 0.75, *p* < 0.001). Higher WRCQ scores were significantly associated with psychological distress, particularly depression, anxiety, somatization, and paranoid ideation. CFA supported the multidimensional structure with five interrelated domains.

**Conclusion:**

The WRCQ is a reliable and valid multidimensional instrument for assessing work-related criticalities in occupational health settings, offering a useful assessment tool for identifying workplace adversities that emerge as predominant.

## 1 Introduction

Work-related stress has become increasingly widespread and serious in recent years. It is widely recognized as a crosscutting, multi-factorial, complex phenomenon, and frequently documented in industrialized countries. According to the latest European report on occupational safety and health (OSH), approximately half of the working population considers work-related stress to be a common issue (1). Similarly, surveys in different national contexts have shown that workers often perceive stress as one of the most relevant occupational risks (2).

Unlike traditional occupational risks, whose effects are typically uniform and easily identifiable, the risks associated with work-related stress are far more heterogeneous. Individual perceptions of, and responses to, stress can differ considerably, even among workers who share the same job tasks and the same environment. This variability makes it especially challenging to establish a clear causal relationship between exposures to stressors, severity of the consequences, and type of harm experienced (3–5). Moreover, cultural and organizational factors may influence how stress is defined, perceived, and assessed, posing additional challenges for the development and cross-cultural application of standardized tools. For example, in Italy the assessment, and even the definition, of work-related stress remains particularly complex, partly due to a social and organizational context that makes it difficult to directly adopt tools developed in other European countries (6). Nevertheless, the phenomenon has been formally recognized as having a significant impact on workers’ health, and the Consolidated Law on Health and Safety at Work (Legislative Decree 81 of April 9, 2008 (7)) has made it mandatory to assess organizational risks that may potentially generate stress.

The need to evaluate these risks is widely recognized in occupational health practice (8, 9). Clinical frameworks usually combine the administration of psycho-diagnostic tests with specialist interviews, with the aim of reconstructing a potential link between negative workplace experiences and the reported psychophysiological consequences. In Italy, for instance, dedicated Centers for work-related stress have adopted this integrated approach, emphasizing the importance of linking organizational factors to individual health outcomes (10).

Many instruments have been developed to identify and measure dynamics commonly described as mobbing or workplace bullying. These include the Leymann Inventory of Psychological Terror [LIPT; Leymann (11) and Ege (12)], one of the earliest psychometric tools on bullying, and the Work Harassment Scale [WHS; Björkqvist et al. (13) and Di Fabio et al. (14)]. The most widely used instrument worldwide is the Negative Acts Questionnaire [NAQ; Einarsen and Raknes (15) and Einarsen and Hoel (16)], currently available in its revised version [NAQ-R; Einarsen and Hoel (17)]. These instruments have significantly contributed to the operationalization of interpersonal hostility dynamics but they are mainly focus on negative acts and harassing behavior.

At the same time, research on psychosocial risks and our systematic clinical observation of recurring patterns of workplace distress suggested that work-related stress is a multi-level phenomenon that emerges from a larger range of interpersonal and organizational adversities (10, 18, 19). For this reason, while the present work fully acknowledges the relevance of stress and bullying-related constructs, it adopts the wider notion of “work-related criticalities.” This conceptualization derives directly from clinical practice: through repeated assessments of workers reporting distress, we consistently observed a set of recurrent “critical” elements in their narratives, such as relational tensions, perceived hostility, organizational inefficiencies, and experiences of professional devaluation, which, although heterogeneous, appeared to co-occur and contribute jointly to the development and maintenance of stress-related conditions. This perspective is in line with a multifactorial perspective consistent with major occupational stress models (e.g., Effort–Reward Imbalance, Job Demand–Control, and transactional approaches), which similarly emphasize the interplay between organizational conditions and subjective appraisal (20–22).

Thus, this terminology was adopted to reflect the complexity and the multifactorial nature of the psychological and organizational processes involved in the experience of work-related stress. This study introduces the Work-Related Criticalities Questionnaire (WRCQ), a self-reported instrument, developed in an Italian center for Occupational Stress and intended to assess/capture the worker’s subjective perception of workplace adversities. Rather than verifying the objective occurrence of reported critical working conditions, this instrument aims to evaluate how workers perceive potential sources of stress, so that it is subsequently possible to better understand how such perceptions may be relevant in relation to psychophysiological outcomes.

Five dimensions describe the main configurations in which workplace criticalities may manifest: interpersonal conflict, understood as a symmetrical and potentially physiological relational dynamic; interpersonal hostility, characterized by asymmetry, directionality of action and risk of victimization; organizational dysfunctions, referring to systemic and widespread organizational problems; organizational constraints, understood as targeted and coercive organizational practices; and professional devaluation, related to processes affecting professional identity and role delegitimization. These dimensions reflect three complementary perspectives from which the phenomenon can be examined, the relational, organizational and professional-identity-related point of view. We conceptually differ them in terms of relational structure (symmetrical versus asymmetrical dynamics), level of analysis (interpersonal versus organizational), and object of impact (working conditions versus professional identity). However, they were not conceived as independent constructs, but as interconnected expressions of a broader and unified domain of workplace adversity, representing different configurations of the same underlying condition.

The questionnaire may be especially relevant in the Italian context, where workplace stress remains a significant and well-documented issue (23), and only a limited number of assessment tools are available, often in partial or preliminary forms (24, 25).

To address these gaps, our aim is to assess the psychometric properties of the WRCQ in a clinical sample of Italian workers, by examining its reliability, validity, multidimensional structure, and associations with psychological outcomes.

## 2 Methods

### 2.1 Participants and setting

This retrospective study examined workers evaluated at the Center for Occupational Stress and Harassment of the Policlinico Hospital of Milan between 2021 and 2025. All data were retrieved from clinical records routinely collected during the multidisciplinary assessment process, which includes occupational medical evaluation and psychological consultation (10, 26). No formal exclusion criteria were applied. Inclusion was based on the availability of a full clinical assessment and completed self-report measures which required sufficient proficiency in Italian to understand and complete the questionnaires. For each patient, sociodemographic information, occupational characteristics, clinical history, and questionnaire data were anonymously extracted. Data collection was conducted in accordance with the Helsinki Declaration and the national law. Because the General Authorization to Process Personal Data for Scientific Research Purposes (Authorization No. 9/2014) declares that ethics approval is not needed for retrospective archive studies that use codes, ethical committee approval was not required.

### 2.2 Development and structure of the WRCQ

The WRCQ was developed within a clinical setting for occupational stress assessment, based on long-term observation of recurrent workplace difficulties reported by workers. Item generation was informed by a structured checklist used over several years at the Center to systematically collect work-related stressors, and was broadly aligned with international recommendations on psychosocial risks and workplace adversity.

Since the development of this questionnaire has always been based on the clinical observation of an Italian sample, particular attention was given to including situations reflecting the specific characteristics of the Italian occupational context, including relevant organizational practices and regulatory aspects. This approach enhances the instrument’s sensitivity to context-specific features that may not be fully represented in standardized tools. The initial structure of the checklist was organized around three broad areas: attacks on the person, attacks on the work situation, and punitive actions. These areas were progressively refined to better represent the different ways in which workplace adversities may manifest in clinical practice, with the aim of improving the interpretability of reported experiences. Following this process, 39 items were finally reorganized into five domains reflecting recurrent patterns observed in the clinical setting: *Interpersonal hostility (IH)* captures experiences of personal isolation, criticism of one’s work, poor communication, and disciplinary actions; *interpersonal conflict (IC)* includes provocations, personal attacks, lack of cooperation, and being ignored in one’s initiatives. *Organizational dysfunctions (OD)* refer to situations in which workers are assigned tasks incompatible with their health status, face excessive workloads, or encounter obstacles in accessing leave or other organizational accommodations. *Professional devaluation (PD)* concerns organizational behaviors that devalue the individual and undermine their professional role. *Organizational constraints (OC),* developed in reference to the “organizational constraints” recognized by INAIL (27), reflect efforts by the employer to limit autonomy, discretion, and creativity, and include more severe scenarios such as demotion, enforced inactivity, or exclusion from training and company projects. The definition and organization of these domains were guided by clinical experience within a multidisciplinary team, including occupational health physicians and psychologists.

Each item is rated on a 3-point scale (never, sometimes, often), scored from 1 to 3, respectively. A total score is calculated by averaging the item scores, resulting in an overall measure of subjectively perceived work-related criticalities, with higher scores indicating a greater degree of workplace adversity (range 1–3). Subscale scores are computed by averaging the items belonging to each specific domain. The full questionnaire, developed in Italian, is provided in the Supplementary material with its English translation.

### 2.3 Additional measures

To support construct validity, we extracted data on: (i) The Symptom Checklist-90-R (SCL-90-R, 90 items (28, 29)), a widely used instrument for assessing psychological distress and symptomatology across multiple domains (somatization, obsessive-compulsive, interpersonal sensitivity, depression, anxiety, hostility, phobic anxiety, paranoid ideation, and psychoticism). (ii) The State–Trait Anger Expression Inventory (STAXI, 44 items (30, 31)), which measures the intensity and expression of anger. (iii) The Italian version of the Negative Acts Questionnaire-Revised (NAQ-R) for a subsample of participants (32). (iv) Sociodemographic and occupational data, including participant’s sex, age, job classification, employment sector, and current work status (e.g., employed, unemployed, dismissed, on sick leave, or on leave of absence). Information regarding ongoing psychotherapy, psychotropic medication use, and the involvement of the occupational health physician was recorded.

### 2.4 Sample characteristics

A total of 317 workers were included in the analysis, of whom 193 (61%) were female and 124 (39%) males. The mean age was 50.5 years (sd = 7.7). Regarding the professional role, the majority were employees (*N* = 215; 68%), followed by manual workers (*N* = 55; 17%), mid-level managers (*N* = 36; 11%), and executives (*N* = 11; 3%). Employment status at the time of assessment was distributed as follows: 225 participants (71%) were actively employed, 49 (15%) on medical leave, 25 (8%) unemployed, and 7 (2%) on leave of absence or administrative suspension. Participants were employed most in services (*N* = 200; 63%), followed by healthcare (*N* = 40; 13%), industry (*N* = 34; 11%), public administration (*N* = 32; 10%), and education (*N* = 10; 3%). A total of 96 participants (30%) reported being in psychotherapy and 210 (66%) reported taking psychotropic medication. The occupational health physician was involved in 89 cases (28%).

### 2.5 Statistical analyses

The internal consistency of the questionnaire was assessed through Cronbach’s alpha. To examine convergent validity, Pearson’s correlations were calculated between the scale and the NAQ-R. Construct validity was explored by testing associations between WRCQ score and psychological symptoms, as measured by the SCL-90-R, and anger expression (STAXI), in line with existing literature. Measurement invariance was assessed to evaluate the equivalence of the scale across groups by conducting subgroup analyses, using Student’s t-tests or one-way ANOVA as appropriate. Confirmatory factor analysis (CFA) was conducted to test the proposed multidimensional structure of the WRCQ. Three alternative models were estimated: a single-order model, a five-factor correlated model, representing the hypothesized sub-dimensions of workplace criticalities, and a second-order factor model, including a general “work criticality” factor above the five sub-dimensions, accounting for shared variance among the subscales, to test whether the five domains could be interpreted as expressions of a broader workplace adversity construct. Analyses were conducted on the polychoric correlation matrix given the ordinal 3-point response scale. We used WLSMV (weighted least squares mean and variance adjusted), recommended for categorical indicators. All analyses were conducted using R software. A *p* value <0.05 was considered as significant.

## 3 Results

Table 1 presents the descriptive statistics of the WRCQ total scale and subscales. The mean total score was 1.8 (sd = 0.4, range 1–2.8). Among the scale dimensions, the highest scores were observed in the domains of IC and PD (both with mean = 2.0), whereas IH showed the lowest mean score (1.7). The mean NAQ-R score was 2.1 (sd = 0.4). The scale demonstrates very good internal consistency, with a Cronbach’s alpha of 0.91 (95% CI 0.90–0.93). Most subscales also showed high internal consistency (alpha >0.7), except for the organizational dysfunctions subscale (0.63). Subgroup analyses, reported in Supplementary Table S1, showed no significant differences, supporting measurement invariance of the scale.

**Table 1:** Summary statistics for scales, including mean, standard deviation, and Cronbach’s alpha.

Work criticalities	Mean ± sd	Cronbach’s alpha (95%CI)
Total scale	1.8 ± 0.4	0.91 (0.90, 0.93)
Interpersonal hostility	1.7 ± 0.4	0.83 (0.80, 0.85)
Interpersonal conflict	2.0 ± 0.5	0.74 (0.70, 0.78)
Organizational dysfunctions	1.7 ± 0.4	0.63 (0.56, 0.69)
Professional devaluation	2.0 ± 0.6	0.70 (0.65, 0.75)
Organizational constraints	1.9 ± 0.5	0.71 (0.66, 0.75)

Looking at the individual items, the situations most frequently reported involved having received provocations intended to cause a loss of control (Item 11, IC), feeling that one’s ideas or proposals were deliberately ignored or diminished (Item 20, IC), and receiving lower performance evaluations than deserved (Item 21, PD). On the other hand, episodes such as physical violence (Item 12, IH), and anonymous threatening calls (Item 14, IH), were reported much less frequently.

Table 2 reports descriptive statistics of the other measures and their correlation with WRCQ. Convergent validity was demonstrated by a high and significant positive correlation between the WRCQ total score and the NAQ-R (r = 0.75, *p* < 0.001). Significant positive correlations were also found between the NAQ-R and all WRCQ subscales, including interpersonal hostility (r = 0.70, *p* < 0.001), interpersonal conflict (r = 0.64, p < 0.001), organizational dysfunctions (r = 0.59, p < 0.001), professional devaluation (r = 0.63, p < 0.001), and organizational constraints (r = 0.54, p < 0.001). On the SCL-90-R, several symptom dimensions, including somatization, obsessive-compulsive symptoms, interpersonal sensitivity, depression, anxiety, and paranoid ideation, showed mean scores above typical clinical cutoffs. STAXI scores indicated generally moderate anger levels, with high state anger and internalized anger. The WRCQ total score was significantly correlated with all the psychological symptoms measured by the SCL-90-R, particularly, depression (0.37), anxiety (0.40), somatization (0.39), paranoid ideation (0.44), supporting the construct validity of the scale. Although the correlations with measures of anger from the STAXI were generally null, there was nonetheless a significant correlation between higher WRCQ scores and higher levels of state anger (0.18) and anger control (0.17).

**Table 2:** NAQ-R, SCL-90-R and STAXI: mean, sd, and correlation coefficients with WRCQ scores.

	Mean ± sd	Pearson’s Correlation					
		TS	IH	IC	OD	PD	OC
NAQ-R	2.1 ± 0.4	0.75***	0.70***	0.64***	0.59***	0.63***	0.54***
SCL-90-R							
Global severity index	1.6 ± 0.7	0.45***	0.40***	0.34***	0.28***	0.30***	0.31***
Somatization	1.6 ± 0.9	0.41***	0.38***	0.32***	0.28***	0.25***	0.23***
Obsessive-compulsive	2.0 ± 0.9	0.40***	0.32***	0.31***	0.31***	0.29***	0.25***
Interpersonal sensitivity	1.3 ± 0.8	0.31***	0.29***	0.27**	0.16**	0.22***	0.19*
Depression	2.1 ± 0.9	0.39***	0.33***	0.27***	0.23***	0.26***	0.31***
Anxiety	1.8 ± 0.9	0.40***	0.37***	0.29***	0.27***	0.26***	0.28***
Hostility	0.9 ± 0.8	0.22***	0.18***	0.16*	0.09	0.14*	0.17**
Phobic anxiety	0.9 ± 0.9	0.39***	0.36***	0.22**	0.29***	0.27***	0.28***
Paranoid ideation	1.9 ± 0.9	0.46***	0.47***	0.43***	0.21**	0.29***	0.30**
Psychoticism	0.9 ± 0.7	0.29***	0.27***	0.22*	0.12*	0.18*	0.18**
STAXI							
State anger	83.2 ± 19.4	0.17**	0.16*	0.15**	0.02	0.18**	0.17**
Trait anger	59.5 ± 30.7	0.001	−0.04	0.01	0.06	0.03	−0.04
Trait anger – temperament	30.6 ± 28.5	−0.01	−0.02	−0.02	0.02	0.01	−0.02
Trait anger – reaction	63.9 ± 27.7	0.03	−0.03	−0.02	0.14	0.12*	−0.03
Anger expression-in	84.7 ± 21.2	0.06	0.02	0.02	0.06	0.05	0.07
Anger expression-out	49.6 ± 29.6	0.001	0.001	0.05	−0.1	0.04	−0.01
Anger control-in	42.3 ± 27.7	0.15**	0.11*	0.12*	0.1	0.13*	0.08
Anger control-out	75.2 ± 27.9	−0.04	−0.05	−0.04	−0.07	−0.05	0.01

*p < 0.05; **p < 0.01; ***p < 0.001. TS, total score.

Confirmatory factor analysis supported the hypothesized multidimensional structure of the WRCQ. The five-factor correlated model, including interpersonal hostility, interpersonal conflict, organizational dysfunctions, professional devaluation and organizational constraints, showed an overall good fit to the data (CFI = 0.954, TLI = 0.951, RMSEA = 0.067, 90% CI 0.063–0.072, SRMR = 0.098). All items loaded significantly on their intended factors, with standardized loadings generally in the moderate-to-high range (approximately 0.35–0.88). However, latent factor correlations were high, suggesting that the five dimensions share common variance. A second-order model was tested, in which a general “work-related criticalities” factor accounted for the covariation among the five first-order factors. This model also demonstrated good fit (CFI = 0.950, TLI = 0.947, RMSEA = 0.057, 90% CI 0.053–0.062, SRMR = 0.077) and was more parsimonious. The second-order factor showed standardized loadings ranging from 0.72 to 0.97, supporting the presence of an overarching construct of work-related criticalities that underlies the specific sub-dimensions. No item showed low (<0.20) or non-significant loadings (Supplementary Table S2), and no cross-loadings were specified. The single-factor model showed a poorer fit to the data than the multidimensional solutions (CFI = 0.936, TLI = 0.932, RMSEA = 0.079, SRMR = 0.105).

## 4 Discussion

This study presented an instrument designed to assess workplace criticalities, which shows good psychometric properties in a clinical sample within the Italian occupational context. First, the scale demonstrated excellent internal consistency, as reflected by Cronbach’s alpha. Second, its convergent validity was confirmed through significant correlations with the NAQ-R, the most widely used instrument for assessing workplace bullying.

Confirmatory factor analysis supported a multidimensional structure while also indicating interrelations among the domains and the presence of a higher-order factor. The relatively high correlations observed among factors suggest that these dimensions frequently co-occur, particularly in clinically referred populations. This suggests that the WRCQ captures both specific dimensions of workplace experiences and a broader construct of workplace adversity.

The scale showed significant correlations with psychological distress, as measured by the SCL-90-R, particularly with symptoms of depression, anxiety, somatization, paranoid ideation, and psychoticism. Similar correlations were observed for each of the five subscales. As hypothesized, the five dimensions can be understood as specific expressions of a broader superordinate domain of work-related criticality, therefore sharing a common psychosocial risk foundation. From this perspective, distinguishing among dimensions is primarily meaningful at the phenomenological and clinical levels, rather than reflecting variations in the intensity of their association with psychological distress. These results align with extensive literature indicating that sustained exposure to workplace bullying and adverse organizational conditions is a significant risk factor for mental health issues (33, 34). Experiences of workplace bullying and organizational dysfunction often damage an individual’s sense of safety, self-esteem, and control. Chronic stress from these situations produces physiological and psychological responses, leading to emotional exhaustion, low mood, and symptoms similar to trauma. Over time, these conditions can reduce coping capacity, increasing vulnerability to anxiety, depression, and physical complaints (35–37).

The significant correlations with state anger and internalized anger expression support the fact that experiences of workplace adversity can contribute to heightened emotional reactivity and potential difficulties in emotion regulation. This suggests that exposure to workplace adversities not only induces psychological distress but also increases emotional reactivity and difficulties in emotion regulation. Individuals who perceive themselves as targeted, devalued, or systematically obstructed may experience persistent frustration and helplessness, emotions that can manifest as either internalized anger (e.g., self-directed blame, rumination or externalized expressions of anger). Such emotional dynamics can further compound interpersonal tensions at work, creating a vicious cycle of conflict and stress (26, 38).

The WRCQ allows not only to detect the overall presence of workplace criticalities but also to identify which specific domains are particularly affected. These domain-specific profiles may provide a useful clinical orientation, helping to identify areas where problematic dynamics may be emerging.

Integrating such an instrument into occupational health assessment may support professionals in obtaining a more structured understanding of perceived workplace criticalities, providing an initial orientation for further evaluation.

This, in turn, enables the implementation of primary, secondary, and tertiary prevention measures. Organizational well-being interventions can yield benefits both for companies, in terms of increased efficiency, productivity, and reduced absenteeism and occupational risks, and for workers, through improved health, motivation, self-esteem, and personal fulfilment (39). These potential applications, however, require further investigation in non-clinical and longitudinal studies.

Some limitations must be acknowledged. First, the sample consisted of individuals already referred for occupational stress or harassment evaluation, potentially leading to higher baseline levels of distress and adversarial workplace experiences than would be observed in the general working population. Thus, while the WRCQ shows good psychometric properties in high-risk samples, future research should also include non-clinical samples to assess the generalizability of the instrument. Second, all measures relied on self-report questionnaires, which may be subject to response biases. Moreover, the assessment is based on subjective perception and does not include external or objective measures of workplace exposure, which may limit the ability to distinguish between perceived and actual working conditions. Third, the retrospective design based on clinical records may limit data quality and confounding. However, this approach allowed the analysis of a large sample. Fourth, given the cross-sectional design and the absence of established normative cut-offs, the present findings support concurrent associations and clinical interpretability rather than screening or predictive applications. Further longitudinal research and the evaluation of sensitivity and specificity would be necessary before considering the instrument for screening purposes.

Despite these limitations, the study presents several strengths. First, the development of the WRCQ was grounded in extensive clinical experience, ensuring that the instrument captures culturally specific and organizationally relevant stressors typical of the workplace context. This context-sensitive approach reflected the need to consider the distinctive features of different labor systems and regulatory environments, which may shape how workplace adversities are experienced and reported. In this perspective, assessment tools such as the WRCQ may benefit from periodic updates to remain aligned with ongoing transformations in the labor market. Second, the WRCQ introduces a multidimensional perspective that extends beyond traditional bullying-focused instruments by incorporating organizational dysfunctions and structural constraints, offering a more complete representation of workplace adversity. Third, the instrument remains concise and easy to administer, enhancing its clinical and organizational applicability. Its capacity to generate domain-specific profiles may support more targeted clinical assessment and hypothesis generation. in occupational settings.

## Data Availability

The raw data supporting the conclusions of this article will be made available by the authors, without undue reservation.
