# Supplementary material for: Assessing work-related criticalities: psychometric properties of a questionnaire developed in an Italian occupational stress center
Source: Front Public Health. 2026 May 8;14:1791989. doi: 10.3389/fpubh.2026.1791989 (PMC13194109; doi:10.3389/fpubh.2026.1791989)
Supplement: Supplementary file 1 [file Supplementary_file_1.docx]

Supplementary Material

*Questionnaire items and corresponding domain*

1. Physical isolation with the inability to communicate with colleagues (IH)
2. Deliberate silence when I enter the room. (IH)
3. Exclusion from recreational activities (coffee breaks, etc.) and social interactions (IH)
4. Refusal to collaborate with me. (IC)
5. Bad-taste jokes. (IC)
6. Offensive comments about my sexual orientation, political or religious views, or my geographical origin. (IH)
7. Attacks on my private life. (IC)
8. Spreading of false rumors. (IC)
9. Behaviors intended to incite other people against me. (IH)
10. I have been subjected to sexual harassment (verbal and/or physical).
11. I have received provocations intended to make me lose control. (IC)
12. I have been subjected to physical violence. (IH)
13. I have received threatening or defamatory letters. (IH)
14. I have received anonymous phone calls. (IH)
15. Various types of monitoring are carried out outside the workplace. (IH)
16. I have been assigned dangerous tasks or tasks unsuitable for my state of health. (OD)
17. Someone has tampered with my workstation and/or damaged some of my personal belongings. (IH)
18. My phone calls and/or my personal mail are intercepted and/or monitored. (IH)
19. I am subjected to continuous criticism that does not reflect reality, with refusal to specify the reasons. (IH)
20. Behaviors are adopted to belittle or ignore my proposals or ideas. (IC)
21. I have received performance evaluations lower than my actual performance. (PD)
22. A collaborator has been assigned to work alongside me without notice, progressively reducing my tasks and responsibilities. (PD)
23. It has happened that my merits were attributed to others. (PD)
24. It has happened that I was blamed for things that were not my fault. (IH)
25. I am assigned meaningless tasks. (PD)
26. I am given tasks that require skills that were not originally foreseen or requested. (OD)
27. I am assigned new tasks without notice, without instructions or tools. (OC)
28. I am suddenly overloaded with work with deadlines that are impossible to meet. (OD)
29. I am contacted during holidays or sick leave without valid reasons. (OD)
30. I am not assigned any tasks. (OC)
31. I am denied the right to carry out my duties and/or the authority necessary to do so. (OC)
32. I am assigned work that is not appropriate to my skills. (OD)
33. I am denied the right to participate in training or professional development courses. (OC)
34. Colleagues or supervisors refuse to communicate with me or use indirect communication (memos, faxes, emails, etc.). (IH)
35. I am excluded from work meetings or company projects. (OC)
36. Excessive and pretextual use is made of medical checks and/or fitness-for-work examinations and/or medical board assessments. (IH)
37. Excessive and pretextual use is made of disciplinary charges, without giving me the opportunity to defend myself. (IH)
38. I have been transferred to locations far from my residence or to inconvenient locations.
39. Without reason, permissions, leave, shifts, or transfers are denied to me or granted only with difficulty. (OD)

**Table S1. Subgroup analyses.**

|  | **TS** | **IH** | **IC** | **OD** | **PD** | **OC** |
| --- | --- | --- | --- | --- | --- | --- |
| **Gender** Male Female | 1.8 (0.4)  1.8 (0.4)  *p=0.77* | 1.7 (0.4)  1.7 (0.4)  *p=0.48* | 2.0 (0.4)  2.0 (0.5)  *p=0.94* | 1.7 (0.4)  1.8 (0.4)  *p=0.56* | 2.0 (0.5)  2.0 (0.6)  *p=0.44* | 1.9 (0.5)  1.9 (0.5)  *p=0.44* |
| **Age** <50y ≥50y | 1.8 (0.3)  1.8 (0.4)  *p=0.55* | 1.7 (0.4)  1.7 (0.4)  *p=0.65* | 2.0 (0.5)  2.0 (0.5)  *p=0.40* | 1.7 (0.4)  1.8 (0.4)  *p=0.21* | 2.0 (0.6)  2.0 (0.6)  *p=0.99* | 1.8 (0.5)  1.9 (0.6)  *p=0.14* |
| **Education** Lower secondary school High school diploma University degree | 1.7 (0.3)  1.8 (0.3)  1.8 (0.4)  *p=0.23* | 1.7 (0.3)  1.7 (0.4)  1.7 (0.4)  *p=0.80* | 1.9 (0.5) 2.0 (0.5)  2.0 (0.5)  *p=0.58* | 1.6 (0.4)  1.8 (0.5)  1.7 (0.4)  *p=0.08* | 1.8 (0.5) 2.0 (0.6)  2.1 (0.6)  *p=0.06* | 1.7 (0.5)  1.9 (0.6)  1.9 (0.5)  *p=0.008* |
| **Job position** Blue-collar worker Middle manager White-collar worker Executive | 1.8 (0.4)  1.9 (0.3)  1.8 (0.4)  1.8 (0.4)  *p=0.69* | 1.8 (0.4)  1.7 (0.3)  1.7 (0.4)  1.6 (0.4)  *p=0.24* | 2.1 (0.5) 2.0 (0.4)  2.0 (0.4)  1.9 (0.5)  *p=0.37* | 1.8 (0.5) 1.7 (0.4)  1.7 (0.4)  1.7 (0.6)  *p=0.88* | 2.0 (0.6)  2.1 (0.5)  2.0 (0.6)  2.0 (0.5)  *p=0.40* | 1.7 (0.6)  2.0 (0.5)  1.9 (0.5)  1.9 (0.6)  *p=0.02* |
| **Sector** Industry Education Public administration Healthcare Services | 1.8 (0.3)  1.9 (0.5)  1.9 (0.4)  1.8 (0.3)  1.8 (0.3)  *p=0.28* | 1.8 (0.4)  1.8 (0.5)  1.8 (0.4)  1.8 (0.3)  1.6 (0.3) *p=0.02* | 2.1 (0.5)  2.1 (0.5)  2.0 (0.5)  2.1 (0.5)  1.9 (0.5) *p=0.06* | 1.7 (0.4)  1.9 (0.5)  1.8 (0.4)  1.8 (0.5)  1.7 (0.4) *p=0.39* | 2.0 (0.5)  2.2 (0.6)  2.3 (0.7)  1.9 (0.5)  2.0 (0.6) *p=0.02* | 1.7 (0.4)  1.9 (0.6)  2.1 (0.6)  1.9 (0.5)  1.9 (0.5)  *p=0.17* |
| **Employment status** Employed Unemployed Sick leave Other | 1.8 (0.4)  1.9 (0.3)  1.9 (0.4)  1.8 (0.4)  *p=0.14* | 1.7 (0.4)  1.8 (0.3)  1.7 (0.4)  1.7 (0.4)  *p=0.19* | 2.0 (0.4)  2.0 (0.4)  2.0 (0.5)  2.1 (0.5)  *p=0.28* | 1.7 (0.4)  1.9 (0.4)  1.8 (0.5)  1.8 (0.5)  *p=0.03* | 2.0 (0.6)  2.2 (0.6)  2.0 (0.6)  2.0 (0.6)  *p=0.42* | 1.9 (0.6)  2.0 (0.6)  1.8 (0.5)  1.7 (0.5)  *p=0.27* |
| **Psychotropic medication** Yes No | 1.8 (0.3)  1.8 (0.4)  *p=0.15* | 1.7 (0.4)  1.6 (0.4)  *p=0.09* | 2.0 (0.5)  1.9 (0.5)  *p=0.13* | 1.8 (0.4)  1.7 (0.4)  *p=0.33* | 2.0 (0.6)  1.9 (0.6)  *p=0.24* | 1.9 (0.5)  1.8 (0.5)  *p=0.19* |
| **Psychotherapy** Yes No | 1.8 (0.3)  1.8 (0.4)  *p=0.17* | 1.6 (0.3)  1.7 (0.3)  *p=0.13* | 1.9 (0.5)  2.0 (0.5)  *p=0.48* | 1.7 (0.4)  1.7 (0.5)  *p=0.50* | 2.0 (0.6)  2.0 (0.6)  *p=0.42* | 1.9 (0.6)  1.9 (0.5)  *p=0.81* |
| **Occupational physician**  Yes No | 1.7 (0.4)  1.8 (0.3)  *p=0.11* | 1.6 (0.4)  1.7 (0.4)  *p=0.11* | 1.9 (0.5)  2.0 (0.5)  *p=0.16* | 1.8 (0.54)  1.7 (0.4)  *p=0.46* | 1.9 (0.6)  2.0 (0.6)  *p=0.10* | 1.8 (0.5)  1.9 (0.5)  *p=0.03* |

**Table S2. Standardized factor loadings of the WRCQ (second-order CFA model)**

|  | **Item** | **Std. loading** |
| --- | --- | --- |
| IH | 1 | 0.554 |
|  | 2 | 0.607 |
|  | 3 | 0.610 |
|  | 6 | 0.317 |
|  | 9 | 0.675 |
|  | 12 | 0.230 |
|  | 13 | 0.359 |
|  | 14 | 0.217 |
|  | 15 | 0.386 |
|  | 17 | 0.460 |
|  | 18 | 0.431 |
|  | 19 | 0.663 |
|  | 24 | 0.563 |
|  | 34 | 0.574 |
|  | 36 | 0.370 |
|  | 37 | 0.488 |
| IC | 4 | 0.618 |
|  | 5 | 0.407 |
|  | 7 | 0.451 |
|  | 8 | 0.577 |
|  | 11 | 0.652 |
|  | 20 | 0.694 |
| OD | 16 | 0.452 |
|  | 26 | 0.601 |
|  | 28 | 0.377 |
|  | 29 | 0.434 |
|  | 32 | 0.612 |
|  | 39 | 0.395 |
| PD | 21 | 0.628 |
|  | 22 | 0.532 |
|  | 23 | 0.649 |
|  | 25 | 0.638 |
|  | 27 | 0.568 |
| OC | 30 | 0.472 |
|  | 31 | 0.641 |
|  | 33 | 0.490 |
|  | 35 | 0.751 |
